# Expectant Mothers’ Reflections on Childhood and Parenting: Cross-Sectional Study

**DOI:** 10.2196/80060

**Published:** 2025-11-07

**Authors:** Bente Prytz Mjølstad, Gunnhild Åberge Vie, Linn Okkenhaug Getz, Mari Hegnes Hansen, Hanna Sandbakken Mørkved, Bjarne Austad

**Affiliations:** 1 General Practice Research Unit, Department of Public Health and Nursing Faculty of Medicine and Health Sciences Norwegian University of Science and Technology Trondheim, null Norway

**Keywords:** pregnancy, adverse childhood experiences, parenting, primary health care, general practice, person-centered care, key questions

## Abstract

**Background:**

The significance of parenting practices for children’s development and health has gained increased attention, aligning with life course perspectives on health. Adverse childhood experiences are widespread and linked to impaired parenting in adulthood. Understanding how expectant parents reflect on their childhoods, and how such reflections can be fostered as part of antenatal care, is essential for supporting healthier caregiving and helping to break cycles of intergenerational adversity.

**Objective:**

This study aimed to explore pregnant women’s perspectives on parenting and sources of support in light of their childhood experiences, including responses to a key question about what they want to pass on to their child.

**Methods:**

A cross-sectional online survey was conducted in 2022 among pregnant women recruited through social media platforms, including Facebook groups and Instagram accounts. The Pearson chi-square test was used to examine associations between the assessment of childhood and 4 parenting-related variables. Free-text responses were analyzed for content, categorized, and quantified.

**Results:**

Among 1402 pregnant women across Norway, 10% (139/1393) reported difficult childhoods. Among them, 28.1% (39/139) felt that their upbringing would significantly influence their parenting, compared to 15.9% (171/1074) of those with good childhoods (*P*<.001). Most participants expressed a desire to pass on positive values. For some, the key question prompted disclosure of difficult childhoods and a strong wish to break with their past. Approximately 10% (139/1389) of the participants reported they lacked someone to turn to for parenting advice. This was more common among those with difficult childhoods (37/139, 26.6%) than those with average (28/176, 15.9%) or good childhoods (74/1067, 6.9%; *P*<.001).

**Conclusions:**

Expectant women’s upbringing shapes their parenting expectations and support needs. Many, especially those with difficult pasts, expect these experiences to affect parenting. These findings highlight the role of intergenerational transmission and the value of inviting such reflection as part of antenatal care to identify support needs.

## Introduction

The growing recognition of the many factors that influence health across the life course [1] has led to increased emphasis on parenting as a key factor in children’s development and well-being. Reflecting this trend, expectant parents are increasingly seeking antenatal education that offers early, realistic insights into parenting, along with access to support and guidance [2]. To foster healthy and supportive family environments, the Norwegian government’s parental support strategy places particular focus on meeting the needs of vulnerable families [3].

Early, unplanned pregnancies, often predisposed by adversity in childhood and adolescence [4], can increase the risk of both socioeconomic disadvantage and medical complications related to pregnancy, birth, and child health [5]. Moreover, parental adverse childhood experiences (ACEs) [6] have been associated with elevated parenting stress, with attachment style identified as a potential mediator [7-10]. Conversely, positive childhood experiences, such as feeling supported and perceiving the family as a safe haven, have cumulative benefits and can buffer the negative effects of adversities [11].

ACEs are prevalent, even in high-income countries. According to Norwegian studies, 4% to 7% of adults report having a difficult childhood, and approximately 20% lacked access to trusted adults during their upbringing [12-14]. ACEs are consistently associated with long-term challenges in health, education, and social functioning [6,15]. In Norway, the cumulative economic burden due to ACEs is estimated to account for approximately 2.7% of the gross domestic product [16], underscoring the critical importance of supportive parenting as a focus for public health promotion.

Reducing the long-term and intergenerational impact of ACEs is essential to prevent the past from becoming the prologue to the unborn child’s future [17]. Traditional programs aimed at preventing child maltreatment have shown limited effects [18], and more effective approaches are warranted. A recent meta-analysis showing associations between expectant women’s reflections about their unborn child and later parent-infant interaction quality indicates the relevance of fostering reflection on future parenting strategies during pregnancy [19].

In clinical settings, discussions about parenting and childhood adversity are often emotionally sensitive and require a thoughtful approach. Many first-time parents feel unprepared, frequently expressing concerns about relationships, parenting, and finances [20,21]. While health care providers are expected to discuss parenthood during antenatal care, how they do so is typically left to their discretion [22]. Experts recommend screening for maternal ACEs during pregnancy. Even if only a few cases may be identified, the potential benefits are substantial [23]. Standardized tools such as the ACE questionnaire can be used [5]. Alternatively, or in addition, a more open, nonjudgmental verbal invitation to reflect on one’s upbringing may foster supportive parenting and help build trust, particularly for those in need of additional support. This study marks an initial step in exploring such an approach within the Norwegian context.

In general practice, key questions are a well-established method for initiating dialogues on complex topics in a noninvasive and reflective manner [24,25]. Rooted in patient-centered communication [24-26], such questions are designed to explore complex and sensitive issues with empathy and openness. Key questions are typically single, open-ended, inviting questions that offer structured support for nuanced conversations in general practice. Grounded in person-centered medicine, humanistic psychology, and the biopsychosocial model, these questions reflect a narrative orientation by inviting patients to share their stories, broaden perspectives, deepen reflection, and inspire meaningful change [24,25,27]. Despite the growing awareness of ACEs and the importance of early intervention, structured, person-centered tools remain limited in routine health care. In emotionally significant contexts such as the transition to parenthood, a thoughtfully framed single item may offer a simple yet meaningful entry point for dialogue and support, consistent with narrative and person-centered approaches in health care [27].

Inspired by the use of key questions, we developed and introduced a single open-ended question for expectant parents: “What do you want to pass on from your childhood?” While a single question may not capture the full nuances of multi-item instruments, it can prompt personal reflection and emotionally relevant responses. Previous studies have explored associations between ACEs and parenting, but few have examined these links during pregnancy in a general population sample using both structured and open-ended questions.

This study aimed to examine pregnant women’s perspectives on parenting and sources of support in light of their childhood experiences. As part of this, we explored how they respond to a reflective key question about what they wanted to pass on to their child. To guide the analysis, we asked the following question: “How are self-reported childhood experiences associated with parenting expectations and perceived support–and what aspects of their upbringing do participants want to pass on to their child?”

## Methods

### Study Setting

In Norway, antenatal care and the public child health program are universal, free of charge, and primarily delivered through primary care services. Under the Regular General Practice Scheme, each citizen is assigned to a regular general practitioner (GP), and most GPs provide antenatal care in collaboration with municipal midwives. The Norwegian Public Child Health Program (for children aged 0-5 years) offers regular checkups, vaccinations, and parental support, mainly delivered by public health nurses in cooperation with GPs. Participation in the program is mandatory. Additionally, informal postnatal groups, organized by public nurses, bring together parents of similarly aged infants to share experiences and offer peer support. These groups often continue independently, fostering community and mutual learning over time. Some municipalities also offer birth and parenting preparation courses, though availability varies and is not universal.

### Study Design, Participants, and Data Collection

Between January and March 2022, pregnant women in Norway who were aged 18 years and above were invited to participate in an open, anonymous, cross-sectional online survey. The questionnaire was distributed using Nettskjema, a data collection platform developed by the University of Oslo, which is user friendly and compatible with a wide range of digital devices. The study was promoted through an introductory video and various social media posts. Recruitment was conducted entirely through social media, leveraging 6 Facebook (Meta Platform) due groups and 5 (Meta Platform) accounts. These platforms had a combined follower base of approximately 109,000, although an overlap between followers was likely. For context, 51,480 women gave birth in Norway in 2022. Following the survey launch on a new platform, all responses were received within 4 days. Data collection for each group was closed after 2 to 4 weeks, as no additional responses were submitted. A total of 1402 women completed the survey, with 62% (n=868) recruited via Facebook and 38% (n=534) via Instagram. The study adhered to the Checklist for Reporting Results of Internet E-Surveys (CHERRIES) [28], ensuring transparency and methodological rigor in online recruitment and data collection. The checklist is attached in the Multimedia Appendix 1.

### The Questionnaire

The questionnaire included up to 57 items, depending on the respondent’s answers, and covered five main areas: (1) demographic information; (2) the pregnancy; (3) antenatal care; (4) sources of advice and information; and (5) expectations for the postnatal period and the parental role including reflections on the respondent’s childhood. Of the total items, 12 were open-ended questions with free-text responses. This paper reports findings related to demographics, pregnancy and antenatal care, sources of advice and support, and parental role. The questionnaire included both multiple-answer options and open-ended questions. The other findings are published separately [29,30]. An English translation of the complete questionnaire is attached in the Multimedia Appendix 2.

As no existing validated questionnaire fully aligned with the broader aims of the study, a new instrument was developed. It incorporated selected validated items from previous surveys, including the Cambridge Worry Scale, and questions on demographics, self-rated health, and childhood experiences from the large population-based Norwegian Trøndelag Health Study (HUNT) [29-31]. The single-item question assessing childhood experiences was originally developed for the HUNT study and has been validated, demonstrating good construct validity in capturing the impact of ACEs on adult quality of life [31]. Additionally, inspired by the concept of key questions, we developed a neutral, open-ended single-item question, “What do you want to pass on from your childhood?” This was done to encourage reflection on their childhood experiences and intergenerational transfer. Questions regarding parenting perspectives and supportive networks were developed specifically for this survey. The survey was pilot-tested among 6 pregnant women and did not result in any changes to the variables used in this analysis. Participants provided informed consent by submitting the completed questionnaire, which included an introductory information letter detailing the study’s purpose, estimated completion time, and the voluntary nature of participation. The survey included a back button for answer revision.

### Data Analysis and Study Variables

Data were analyzed using SPSS Statistics (IBM Corp). Descriptive statistics were calculated for the total sample and relevant subgroups. For analysis, we merged the assessment of a very good or good childhood into good and a difficult or very difficult childhood into difficult. Similarly, we merged very great or great extent into great extent and small or not at all into small extent for the inquiry about their thoughts and feelings regarding becoming a parent and how well they felt prepared through antenatal care. The Pearson chi-square test was used to examine associations between the assessment of childhood and the 4 questions concerning influence on raising children, preparation for parenthood, views on becoming a parent, and having someone to seek advice from. Most respondents answered all questions; data on childhood experiences were missing for 9 of 1402 (0.6%) participants. We performed complete case analyses and excluded those with missing responses from each analysis. We analyzed all free-text responses for content, categorized them, and counted the number of responses in each category. The open-ended key question was designed to encourage reflection rather than to identify ACEs directly. Responses were categorized thematically, focusing on values and experiences participants wished to pass on. While coding, we did not systematically assess the presence of ACEs; some responses were interpreted as reflecting adverse experiences, such as neglect, abuse, or instability, based on explicit language used by participants. We additionally used Pearson chi-square tests to assess whether responding to the open-ended key question was associated with demographic characteristics or assessment of own childhood.

### Ethical Considerations

A remit assessment was sent to the Central Norway Regional Committee for Medical and Health Research Ethics, which determined that a formal application was not necessary (reference number 267956). The Norwegian Center for Research Data approved the study (reference number 134424), confirming that no direct or indirect personal identifiers were processed, including no use of cookies or collection of IP addresses. All methods were carried out following relevant national and institutional guidelines and regulations. Informed written consent was obtained from all participants.

Participation was voluntary, and participants could withdraw at any time without providing a reason. No compensation was offered for participation. Data were stored securely on encrypted servers with access restricted to the research team.

## Results

### Overview

A total of 1402 pregnant women, geographically distributed across all regions of Norway, participated in the survey; the demographic characteristics are presented in Table 1.

**Table 1:** Demographics and characteristics of the study population (N=1393-1402).

Variables		Participants, n (%)
**Age (y)**		
	<25	119 (8.5)
	25-37	1226 (87.4)
	>37	57 (4.1)
**Parity**		
	Primipara	722 (51.5)
	Multipara	680 (48.5)
**Gestational age^a^**		
	First trimester	99 (7.1)
	Second trimester	776 (55.4)
	Third trimester	525 (37.5)
**Living with a spouse or partner**		
	Yes	1369 (97.6)
	No	33 (2.4)
**Education**		
	Lower secondary school	28 (2)
	Upper secondary school	315 (22.5)
	College or university (<4 y)	495 (35.3)
	College or university (**≥**4 y)	564 (40.2)
**Residence^b^**		
	Northern Norway	129 (9.2)
	Central Norway	230 (16.4)
	Western Norway	323 (23.1)
	Eastern Norway	631 (45.1)
	Southern Norway	87 (6.2)
**Size of municipality^b^**		
	Rural area (<5000)	270 (19.3)
	Town (5000-20,000)	331 (23.6)
	City (>20,000)	800 (57.1)
**Birthplace**		
	Norway	1336 (95.3)
	Outside Norway	66 (4.7)
**Cultural background^c^**		
	Norwegian	1332 (95.2)
	Not Norwegian	15 (1.1)
	Multicultural	52 (3.7)
**Childhood^d^**		
	Very good	579 (41.3)
	Good	497 (35.4)
	Average	178 (12.7)
	Difficult	102 (7.3)
	Very difficult	37 (2.6)

^a^Missing values: n=2.

^b^Missing value: n=1.

^c^Missing values: n=3.

^d^Missing values: n=9.

### Perceptions of Parenthood in Light of Childhood Experiences

**Figure 1:**
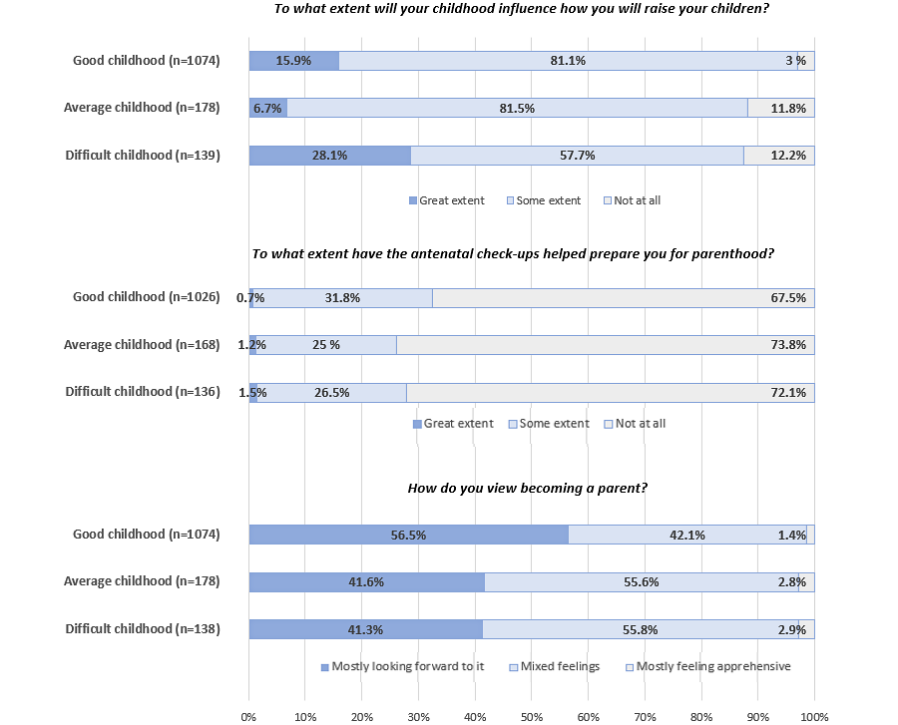
To what extent pregnant women reported that their childhood would influence how they would raise their children, whether the antenatal visits had prepared them for parenthood, and how they viewed becoming a parent, according to how they viewed their own childhood.

The majority (1076/1393, 77.2%) reported their childhood as very good or good, while 10% (139/1393) described it as difficult or very difficult. Figure 1 shows to what extent these women reported that their childhood would influence their parenting, whether antenatal visits prepared them for parenthood, and their views on becoming a parent. Of those with a difficult childhood, 28.1% (39/139) said their upbringing would influence their parenting to a great extent. In comparison, 6.7% (12/178) of the participants with average childhoods and 15.9% (171/1074) of the participants with good childhoods said their upbringing would influence their parenting to a great extent (*χ*^2^_4_=69.0, *P*<.001). Most participants (921/1337, 68.9%) claimed that antenatal checkups had not prepared them for parenthood, with minor differences based on childhood evaluation (*χ*^2^_4_=5.2, *P*=.27). First-time mothers reported this more often than those who had given birth before (506/696, 72.7% vs 415/641, 64.7%, *P*=.007, not shown in Figure 1). Half (740/1401, 52.8%) of the participants were looking forward to becoming parents, while nearly half (635/1401, 45.3%) had mixed feelings, and a small minority group (24/1401, 1.7%) felt apprehensive. More women with a good childhood (607/1075, 56.5%) looked forward to parenthood compared to those with average (74/178, 41.6%) or difficult (57/138, 41.3%) childhoods (*χ*^2^_4_=23.4, *P*<.001). There were no differences based on whether they were first-time mothers or not (*P*=.43).

### Supportive Networks

In total, 10% (139/1389) of respondents reported not having anyone to seek advice from about the parental role, while 90% (1250/1389) had at least 1 person. The proportion who had someone to consult was larger among those with a good childhood (993/1067, 93.1%) compared to those with an average childhood (148/176, 84.1%) and those with a difficult childhood (102/139, 73.4%; *χ*^2^_2_=60.3, *P*<.001).

Among the 826 (N=1402, 58.9%) women who answered an open-ended question on sources for advice, most mentioned family members and friends, with multiple sources reported. Women with a good childhood more frequently cited family members (574/648, 88.6%) compared to those with a difficult childhood (39/78, 50%). Conversely, women with a difficult childhood more often mentioned family-in-law (23/78, 30%) or health personnel (22/78, 28%) compared to those with a good childhood (95/648, 14.7% and 134/648, 20.7%) when asked whom they would seek advice from regarding parenting.

### “What Do You Want to Pass on From Your Childhood?”

Approximately half (669/1402, 47.7%) of the participants responded to the open-ended key question, “What do you want to pass on from your childhood?” Most described positive experiences, such as safety (n=205, 30.6%), love (n=195, 29.1%), care (n=108, 16.1%), openness (n=60, 9%), and being “a present parent” (n=50, 7.5%; ie, being emotionally and physically available, as opposed to being absent).

However, among a smaller group of those who responded (40/669, 6%), the question led to the opposite response; they wrote about what they did not want to pass on, citing negative experiences such as abuse, neglect, and instability. Some participants strongly distanced themselves from their upbringing. A few respondents answered the question with a single word, “nothing,” while others provided more detailed reflections, stating that their childhood would serve as a template for what they did not want for their child (Textbox 1).

**Selected responses to the open-ended question, “What do you want to pass on from your childhood?”**

- “I’ve had an upbringing marked by neglect, so I know very well how I DON’T want to be a parent, and I’ve formed a clear picture of how I want to do the opposite!” [Participant 381]
- “I know what I shouldn’t carry forward from a turbulent upbringing. What I do know is that it should be open to everything and filled with love and care.” [Participant 374]
- “My upbringing serves as a template for what I don’t want for my child.” [Participant 396]
- “I haven’t grown up in safety, so I need to provide my children with that. Stable relationships; I experienced how important it is for development, so I’m taking it with me. Be strong and steady in interactions with others is important for me, even though I wasn’t met that way as a child.” [Participant 183]
- “Minimal exposure to neglect and a violent father. Good and close care from grandparents, presence, and availability.” [Participant 275]
- “The opposite. My child should avoid moving around, having multiple stepfathers, and attending different schools. It should have security and stability with me and the child’s father.” [Participant 220]
- “My child should never experience having their parent involuntarily committed to psychiatric care or something similar. It should never almost lose a parent due to attempted suicide.” [Participant 236]
- “What was good, and, above all, do the opposite of what my parents failed at during my upbringing.” [Participant 229]

### Suggestions for Parenting Support in Antenatal Care

When participants were asked an open-ended question about how the health care system could best support parenting as part of antenatal care, responses varied widely. Approximately one-fourth of the total sample (334/1402, 23.8%) responded. Among them, several participants (110/334, 32.9%) requested more information—either in general or about specific topics (ie, breastfeeding, mental health, and partner relationships)—or access to parenting courses. Others (32/334, 9.6%) emphasized the need for increased dialogue or more personalized follow-up. Greater involvement of the partner was also explicitly requested by 19 (5.7%) participants, and an increase in the number of or longer antenatal checkups was wanted by 18 (5.4%) participants.

## Discussion

### Principal Findings

In this large, digitally recruited sample of pregnant women in Norway, approximately 1 in 10 participants reported having had a difficult or very difficult childhood. Among these, 28.1% (39/139) indicated that their upbringing would strongly influence their approach to parenting, compared to 15.9% (171/1074) of those with a good childhood. Approximately 10% (139/1389) of all respondents reported having no one to turn to for parenting advice, a situation more common among those with difficult childhoods.

Women with positive childhood experiences frequently cited family members as sources of support. Those with difficult childhoods more often mentioned in-laws and health professionals, although some also named specific relatives. Most participants responded to the open-ended key question, “What do you want to pass on from your childhood?” by expressing a desire to transmit positive values and experiences. However, for a smaller group of participants (6%), the question acted as an evocative prompt for disclosing difficult or traumatic aspects of their upbringing, often accompanied by a strong desire to break intergenerational patterns.

Participants also offered suggestions for improving support in antenatal care, including greater involvement of partners, enhanced postnatal follow-up, and more direct conversations about parenting. These findings suggest that the open-ended key question holds promise as a simple yet meaningful tool for initiating reflective and supportive dialogue in routine antenatal care.

### Findings in Relation to Comparable Studies

One out of 10 participating pregnant women reported their childhood to have been difficult or very difficult. This is comparable to another Norwegian study by Haugland et al [13], where 10% of the women assessed their childhood as difficult or very difficult in response to the same question. The proportion of adults reporting the same in the HUNT study has been somewhat lower (4.1%-4.4%) but relatively stable over the last 2 decades [12,14]. Internationally, studies involving adults from various countries found that approximately 1 out of 4 had experienced at least 1 ACE [32,33].

The observed association between reporting a difficult childhood and indicating that their upbringing might significantly influence how they would raise their child aligns well with studies suggesting that the foundation for the parental role is established in one’s upbringing. Furthermore, parents’ ACEs can impact their parenting style in various ways; unresolved emotional issues can affect their ability to respond to their children’s needs and create difficulties in forming secure attachment with their children [8,34].

One out of 10 respondents stated that they had no one to seek advice from regarding their parenting role, while 90% (1250/1389) had at least 1 person. This proportion was significantly lower among pregnant women who had a difficult childhood, and when asked whom they would seek advice from regarding parenting, they mentioned others outside their immediate family, such as in-laws and health care professionals, more frequently. This can be seen in light of previously presented figures from the HUNT study, where 21% reported growing up without a reliable person to seek support from [14]. These women might need more professional advice and guidance from health care personnel. Importantly, nearly 70% (921/1337) of respondents reported that the antenatal checkups had not helped prepare them for the parental role, regardless of their childhood background. This points to a broader potential for improvement in how health care services support expectant parents. Furthermore, only 1 in 4 respondents with a difficult childhood mentioned health care professionals as a source of importance, suggesting that vulnerable groups may not fully recognize or access the help available to them.

Knowledge about the childhood experiences of expectant parents can provide valuable insights for health care providers, allowing them to tailor follow-up and advice about the parental role during antenatal care, as part of preventive health care in public child health centers. Identifying resources within the pregnant women’s networks is crucial, and health care personnel can also take initiatives to strengthen support systems such as postnatal groups.

Participants in our study also shared suggestions for greater involvement of partners. This aligns with a study by Solberg and Glavin [35], which demonstrated that many Norwegian fathers feel sidelined in antenatal care and the public child health care program and express a desire for more direct engagement and recognition of their caregiving role. Despite policy efforts to promote equality in parenting and family-centered services, structural and cultural barriers continue to limit fathers’ participation [3,35].

Our study demonstrated that a digitally delivered, open-ended, and neutrally framed question encouraged expectant parents to reflect on their upbringing and, in some cases, disclose ACEs, a topic often considered sensitive. This approach appeared especially evocative for women with difficult childhoods, introducing a rare and safe space to express concerns and articulate a desire to break negative intergenerational patterns. The phrase thereby appears promising as a key question in the sense introduced earlier, that is, a meaningful entry point for person-centered dialogues [24,26]. With further development and validation, it could become part of a communicative approach aimed at identifying support needs and fostering reflective conversations in antenatal care.

### Methodological Strengths and Limitations

This study has several strengths aligned with the CHERRIES [28], including a large sample size and the use of an anonymous and accessible online format that likely encouraged honest responses, even to sensitive questions. The questionnaire combined previously validated items with newly developed questions, which were pilot-tested for clarity and relevance.

The sample was broadly comparable to the general population of pregnant women in Norway, although there was a slight overrepresentation of first-time mothers, participants from central Norway, and those with higher education (Multimedia Appendix 3). Only 4.7% (66/1402) of participants were born outside Norway, limiting the generalizability of the findings. Missing data were minimal and considered negligible for the interpretation of results.

Participants were recruited through digital platforms and social media, which provided efficient access to the target population. However, this approach may have introduced self-selection bias, a recognized limitation in web-based surveys [28]. Due to the nature of social media recruitment, a traditional response rate could not be calculated.

Despite these limitations, the large and diverse sample enhances the reliability of findings. The broad scope of the questionnaire reduces the likelihood that participation was driven solely by interest in parenting or childhood experiences.

Considering both strengths and limitations, we regard the inclusion of a new single-item open question in the digital survey as a methodologically promising approach. Although only half (699/1402, 47.7%) of the patients responded, and response rates were higher among women with greater awareness of their upbringing and those with higher educational attainment, those who engaged provided meaningful reflection and elicited distilled insights into complex personal experiences. These findings suggest its applicability as a key question in clinical settings, such as antenatal care and child welfare services. Overall, the study provides valuable insights into how digital tools can support the development of antenatal care and highlights the potential of social media for recruiting and engaging expectant parents in research on this matter.

### Conclusions

This study underscores how pregnant women’s childhood experiences shape their expectations and needs regarding parenting. Many, particularly those with difficult pasts, anticipated that these experiences would influence their parenting approach. While some reported a lack of adequate support, many also expressed a strong desire to pass on positive values, reflecting both vulnerability and resilience. On the basis of a digital survey, our findings suggest that reflective key questions may be a promising tool for exploring intergenerational influences among parents. Future research should focus on adapting and testing such questions in clinical practice to support early identification and guide tailored interventions.

## Appendix

Multimedia Appendix 1 CHERRIES checklist.

Multimedia Appendix 2 English questionnaire.

Multimedia Appendix 3 Birth demographics for Norway in 2021.

## Abbreviations

ACE: adverse childhood experience
CHERRIES: Checklist for Reporting Results of Internet E-Surveys
GP: general practitioner
HUNT: Trøndelag Health Study

## Data Availability

The datasets used and analyzed during this study are available from the corresponding author upon reasonable request.
