# Supplementary material for: Expectant Mothers’ Reflections on Childhood and Parenting: Cross-Sectional Study
Source: JMIR Form Res. 2025 Nov 7;9:e80060. doi: 10.2196/80060 (PMC12639342; doi:10.2196/80060)
Supplement: Multimedia Appendix 2 [file formative_v9i1e80060_app2.docx]

### **Appendix 2**

**The questionnaire, translated into English**

**Part 1: Background Information**

**How old are you?**

Under 25

25-37 years

Over 37 years

**What is your highest completed education?**

Primary school

High school

College/University - less than 4 years

College/University - 4 years or more

**What was your main activity before the current pregnancy?**

Employed

Student

Apprentice

Unemployed (but looking for a job)

On welfare benefits, unable to work

Housewife with housework/caring responsibilities

**In this pregnancy have you been or are you still...**

On sick leave? yes/no

Received maternity allowance? yes/no

**Do you live together with someone?**

No, I live alone

Yes, spouse/cohabitant/partner

Yes, other people 18 years of age or older

If yes: How many (other than cohabitant/spouse) aged 18 or older do you live with?

Yes, other people under 18 years old

If yes: How many people under the age of 18 do you live with?

**Is your financial situation such that you can handle an unexpected bill of NOK 10,000 (e.g., for dental care or a repair?**

Yes

No

Don't know

**Where were you born?**

Norway

Other country

**What is your cultural background?**

Consider myself Norwegian

Do not consider myself Norwegian

Consider me as bi/multicultural

**What is your mother tongue?**

Norwegian

Another Nordic language

Another European language

Another non-European language

**What part of the country do you live in?**

Northen Norway

Central Norway

Western Norway

Eastern Norway

Sothern Norway

**How far is it to the nearest hospital/maternity ward from where you live? (Time driving a car)**

Under 1 hour

1-2 hours

More than 2 hours

**How will you define the size of the residence you live in (numbers of citizens)?**

Village/small town (less than 5,000 inhabitants)

Medium-sized town (5 000 – 20,000 inhabitants)

City (more than 20,000 inhabitants)

**Part 2: About the pregnancy**

**How many times have you been pregnant, including your current pregnancy?**

1

2-3

More than 3

**Have you given birth before? (including any caesarean section)**

Yes

No

**How far along are you in this pregnancy?**

1st trimester (from the last menstruation - week 12)

2nd trimester (from week 13 - week 28)

3rd trimester (from week 29 - birth)

**BEFORE you became pregnant, how would you characterize your own health?**

Very good

Good

Not so good

Poor 

**How is your health NOW?**

Very good

Good

Not so good

Poor 

**BEFORE you became pregnant, how were you feeling emotionally/mentally?**

Very good

Good

Not so good

Poor 

**How are you emotionally/mentally NOW during the pregnancy?**

Very good

Good

Not so good

Poor 

**To what extent have you been worried about the following in your pregnancy so far?**

Possible answers: Not at all - To a small extent - To some extent - To a large extent - To a very large extent

1. Own health
2. Consumption of certain foods (that pregnant women should be cautious with)
3. Alcohol consumption (before or after you knew you were pregnant)
4. Physical exercise
5. Relationship and/or family situation
6. Employment
7. Your family's financial situation
8. Miscarriage/loss of the baby
9. That there should be something wrong with the baby
10. The birth itself
11. That you or your partner will not master parenting role

**Are there other things you've worried about?** Avoid information that can identify you personally

**Part 3: Antenatal Care - Information and Advice**

**Have you attended antenatal check-ups so far in your pregnancy?**

Yes, all of them

Yes, most of them

No

Not relevant (haven’t had the first check-up yet)

**Who do you see for antenatal check-ups? (Select all that apply)**

(This item will only appear if the option "Yes, all" or "Yes, most" is selected in the question "Have you attended antenatal check-ups so far in your pregnancy?")

General Practitioner

Midwife

Gynecologist

Others

**What do you think is the reason you have not attended check-ups?**

(This item will only appear if the option "Yes, most" or "No" is selected in the question "Have you attended antenatal check-ups so far in your pregnancy?")

**How satisfied are you overall with the antenatal check-ups in your current pregnancy?**

Not at all

To a small extent

To some extent

To a large extent

To a very large extent

Not relevant (haven’t been there yet)

**To what extent do you feel that the information you have received so far in your pregnancy has been tailored to your needs and concerns?**

Not at all

To a small extent

To some extent

To a large extent

To a very large extent

**Have you had the opportunity to talk to healthcare professionals about the issues/concerns you are interested in/have experienced?**

Not at all

To a small extent

To some extent

To a large extent

To a very large extent

**What do you like to have talked more about with healthcare professionals?**

(This item will only appear if the option 'Not at all', 'To a small extent' or 'To some extent', is selected in the question 'Have you had the opportunity to talk to healthcare professionals about the things/issues you are concerned with/have experienced?')

**Do you feel that you have received sufficient information from healthcare professionals so far in your pregnancy?**

Not at all

To a small extent

To some extent

To a large extent

To a very large extent

**Have you received information/advice at antenatal controls that has made you worried afterwards?**

Yes

No

If yes: **What advice/information has made you worried?**

**Have you had an early ultrasound (i.e. before the routine ultrasound in week 17-19)**

Yes

No

If yes: **Do you feel that you received sufficient information before the early ultrasound?**

Yes

No

**Have you had the routine ultrasound (screening in weeks 17-19)?**

Yes

No

If yes: **Do you feel that you received sufficient information before the ultrasound check-up?**

Yes

No

**Have you attended childbirth preparation courses?**

Yes

No, but plan to attend

No, do not plan to participate

**Part 4: Sources of Information**

**Who do you turn to for advice and support during the pregnancy?**

Possible answers: Not at all - To a small extent - To some extent - To a large extent – To a very large extent

1. Family member
2. Friends
3. Health professionals
4. Other pregnant women that I know
5. People online that I don't know personally
6. Others

**Are there others you seek advice from?** Avoid information that can identify you personally

**To what extent do you feel that family, friends, and/or acquaintances have had opinions or interfered with your pregnancy in ways you have experienced as intrusive or uncomfortable?**

Not at all

To a small extent

To some extent

To a large extent

To a very large extent

**Where do you seek information if you want to learn more or have questions about your pregnancy?** (Grade according to how frequent you have used the different sources)

Possible answers: Not at all - To a small extent - To some extent - To a large extent – To a very large extent

1. Healthcare personnel in the public health service (midwife/doctor)
2. Healthcare personnel in a private or other setting (courses, etc.)
3. Public quality assured websites (e.g. Norwegian Institute of Public Health, ‘Helsenorge’)
4. Discussion forum
5. Facebook groups
6. Influencers, blogs
7. Podcasts
8. Apps
9. Friends
10. Family
11. Learned in own education
12. Other sources like Snapchat, Instagram, books

**Are there other sources of information besides the above ones you have used when searching for information in connection with your pregnancy?**

**How often would you estimate that you retrieve information/read about pregnancy/birth or parenting?**

Several times daily

Daily

Several times weekly

Less than 1 time per week

Avoids seeking information

**Part 5: Thoughts About the Postpartum Period and Own Parenting**

**How do you feel about becoming a parent?**

Mostly looking forward to it

Mixed emotions - both dreading and looking forward to it

Mostly dreading it

Other

**What else do you think about becoming a parent?** Answer with key words.

(This item will only appear if the "Other" option is selected in the question "How do you reflect on becoming a parent?")

**To what extent have you had concerns about the following conditions AFTER childbirth during pregnancy?**

Possible answers: Not at all - To a small extent - To some extent - To a large extent - To a very large extent

1. The relationship to your partner, or relationship to the other parent if you do not live together
2. Economic situation
3. Employment
4. Physical changes of the body (in connection with childbirth)
5. Not being able to breastfeed
6. Not coping with motherhood
7. The environment in which the child grows up
8. Not having access to support and help from others during the infant and toddler period

**Have you had any concerns other than those mentioned?**

**To what extent do you think your own upbringing will influence how you will raise your own children?**

Not at all

To a small extent

To some extent

To a large extent

To a very large extent

**What do you want to bring on from your own upbringing in the upbringing of your own children? (Keywords).**

**Do you have someone you can seek advice from regarding parenting?**

Yes

No

If yes: **Who can you seek advice from?**

**When you are thinking about your childhood/upbringing, how would you describe it?**

Very good

Good

Average

Difficult

Very difficult

**To what extent do you feel that antenatal check-ups so far have helped prepare you for childbirth and the postpartum period?**

Not at all

To a small extent

To some extent

To a large extent

To a very large extent

Not applicable

**To what extent do you feel that antenatal check-ups so far have helped prepare you for parenting?**

Not at all

To a small extent

To some extent

To a large extent

To a very large extent

Not applicable

**Feel free to write more about how you think the health service and antenatal care can best help support parenting as part of prenatal care**

**Feel free to write more about how you want antenatal care to be in the future**

**Thank you for participating!**

Feel free to write down comments on the questionnaire, or more about your experiences here
