# Supplementary material for: Expectant Mothers’ Reflections on Childhood and Parenting: Cross-Sectional Study
Source: JMIR Form Res. 2025 Nov 7;9:e80060. doi: 10.2196/80060 (PMC12639342; doi:10.2196/80060)
Supplement: Multimedia Appendix 3 [file formative_v9i1e80060_app3.docx]

### **Appendix 3**

*Demographics characteristics of the pregnant women who gave birth in Norway in 2021, based on publicly available data from the Norwegian Medical Birth Registry (*<http://statistikkbank.fhi.no/mfr/>)

| **Variables** |  | **n** | **%** |
| --- | --- | --- | --- |
| **Age**  (n = 55 892) | < 25  25-34  35-39  > 39 | 4 261  39 183  10 238  2 210 | 7.6  70.1  18.3  4.0 |
| **Parity**  (n = 55 892) | Primipara  Multipara | 23 751  32 141 | 42.5  57.5 |
| **Marital status**  (n = 55 892) | Married/cohabiting  Other | 52 762  3 123 | 94.4  5.6 |
| **Residence**  (n = 56 671) | Northern Norway*  Central Norway*  Western Norway*  Eastern Norway*  Southern Norway* | 4 727  7 641  12 450  24 909  6 755 | 8.3  13.5  22.0  44.0  11.9 |
| **Maternal birth place**** | Norway  Other country |  | 73,4  26,6 |

** Northern Norway includes counties Finmark, Troms and Nordland. Central Norway includes counties Trøndelag and Møre og Romsdal, Western Norway includes counties Vestland and Rogaland, Eastern Norway includes counties Viken, Oslo and Innlandet, and Southern Norway includes counties Telemark, Vestfold and Agder.*

***Numbers from; Ottesen HS, Sørbye IK, Lindskog BV, Vangen S, Sundby J, Owe KM. Caesarean sections among immigrant women with different levels of education. Tidsskr Nor Laegeforen. 2022, 21;142(17).*
